# MALDI Q-TOF CID MS for Diagnostic Ion Screening of Human Milk Oligosaccharide Samples

**DOI:** 10.3390/ijms15046527

**Published:** 2014-04-16

**Authors:** Marko Jovanović, Richard Tyldesley-Worster, Gottfried Pohlentz, Jasna Peter-Katalinić

**Affiliations:** 1 Department of Biotechnology, University of Rijeka, Radmile Matejčić 2, Rijeka 51000, Croatia; 2 Waters Corporation, Stamford Avenue, Altrincham Road, Wilmslow SK9 4AX, UK; E-Mail: richard_tyldesley-worster@waters.com; 3 Institute for Hygiene, University of Muenster, Robert-Koch-Strasse 41, Muenster D-48149, Germany; E-Mail: pohlentz@uni-muenster.de; 4 Institute for medical Physics and Biophysics, University of Muenster, Robert-Koch-Strasse 31, Muenster D-48149, Germany; E-Mail: jkp@uni-muenster.de or jasnapk@biotech.uniri.hr

**Keywords:** human milk oligosaccharides, MALDI Q-TOF (matrix-assisted laser desorption/ionization quadrupole-time-of-flight), MS (mass spectrometry), CID (collision-induced dissociation), diagnostic ion MS, *de novo* sequencing

## Abstract

Human milk oligosaccharides (HMO) represent the bioactive components of human milk, influencing the infant’s gastrointestinal microflora and immune system. Structurally, they represent a highly complex class of analyte, where the main core oligosaccharide structures are built from galactose and *N*-acetylglucosamine, linked by 1–3 or 1–4 glycosidic linkages and potentially modified with fucose and sialic acid residues. The core structures can be linear or branched. Additional structural complexity in samples can be induced by endogenous exoglycosidase activity or chemical procedures during the sample preparation. Here, we show that using matrix-assisted laser desorption/ionization (MALDI) quadrupole-time-of-flight (Q-TOF) collision-induced dissociation (CID) as a fast screening method, diagnostic structural information about single oligosaccharide components present in a complex mixture can be obtained. According to sequencing data on 14 out of 22 parent ions detected in a single high molecular weight oligosaccharide chromatographic fraction, 20 different oligosaccharide structure types, corresponding to over 30 isomeric oligosaccharide structures and over 100 possible HMO isomers when biosynthetic linkage variations were taken into account, were postulated. For MS/MS data analysis, we used the *de novo* sequencing approach using diagnostic ion analysis on reduced oligosaccharides by following known biosynthetic rules. Using this approach, *de novo* characterization has been achieved also for the structures, which could not have been predicted.

## 1. Introduction

Human milk oligosaccharides (HMO) are the third most abundant type of component in human milk [1]. Their core structure is biosynthesized by adding *N*-acetyllactosamine units to the single lactose core at the reducing end. Apart from variable branching patterns, even a more significant layer of structural diversity is provided by extensive fucosylation. Recent studies have shown that HMO consist of around 200 oligosaccharide structures, including both neutral and negatively charged structures [2–4]. Their most understood function is the interaction with infant’s gut microflora, stimulating the growth of probiotic bacteria [5,6] and providing immunological benefits to the infant [7]. Based on animal studies, a role of sialylated oligosaccharides in brain development has been postulated [8,9]. In recent studies, milk oligosaccharide patterns from women delivering preterm and at term were compared [10], focusing on differences in fucosylation patterns and lacto-*N*-tetraose (LNT) abundance. In spite of significant advances in the analytical techniques used for oligosaccharide structure elucidation over the past two decades [2–4,11–19], all individual components of HMO cannot be fully revealed.

In addition to the complex nature of HMO biosynthesis, additional variety in HMO structures can arise from chemical procedures carried out during sample preparation steps or due to biological enzymatic activity. To these belongs also the chemical procedure of mild acid hydrolysis in order to remove fucose and sialic acid residues [20]. This may be necessary to simplify a detailed structural analysis of the oligosaccharide core structure. It has also been found that sugars on the nonreducing terminus of the oligosaccharide are susceptible to cleavage by glycosidases in breast and during the storage of milk, although this degradation was found to be modest [21]. Other experimental parameters and biological specificities of the milk donor may influence HMO composition. For targeted (data-dependent acquisition or DDA) MS analysis methods, preliminary characterization of the sample is necessary. Matrix-assisted laser desorption/ionization (MALDI) MS is the method of choice for oligosaccharide screening, in particular in the context of high-throughput options. In most cases, MALDI-MS fingerprints of glycan mixtures display a singly charged ionic signal per component, which tends to simplify spectral interpretation in comparison to electrospray ionization (ESI). For most experiments conducted on glycans, ubiquitous sodium ions from glass and other sources attach to glycan molecules to form [M + Na]^+^ ions. For direct measurements on neutral glycans, [M + Na]^+^ abundances (peak area) may be compared in a semi-quantitative way within the same sample [22]. Combined with collision-induced dissociation (CID) available on quadrupole-time-of-flight (Q-TOF) mass spectrometers, it can quickly provide sufficient information for subsequent, more detailed MS analyses for a large number of precursor ions.

For large molecular weight HMO, which can possess more complex branching sites, as well as multiple fucosylation sites, the structures are predominantly difficult to define, also due to isobaric mixtures. This is frequently solved by the application of liquid chromatography (LC) protocols, which allow isomer separation prior to MS analysis [2,3]. More direct is the MALDI Q-TOF CID *de novo* approach. We show that the already known oligosaccharide structures can be assigned according to specific diagnostic ions in combination with the HMO biosynthetic rules, but also, novel structure types can be proposed *de novo*. The additional advantages of the MALDI Q-TOF MS analysis are the sensitivity, speed, high dynamic range and ability to analyse data in more detail on the same sample spot once the preliminary data analysis is completed.

## 2. Results and Discussion

### 2.1. MALDI TOF (Matrix-Assisted Laser Desorption/Ionization Time-of-Flight) Mapping of Complex Oligosaccharide Mixtures

**Figure 1.**
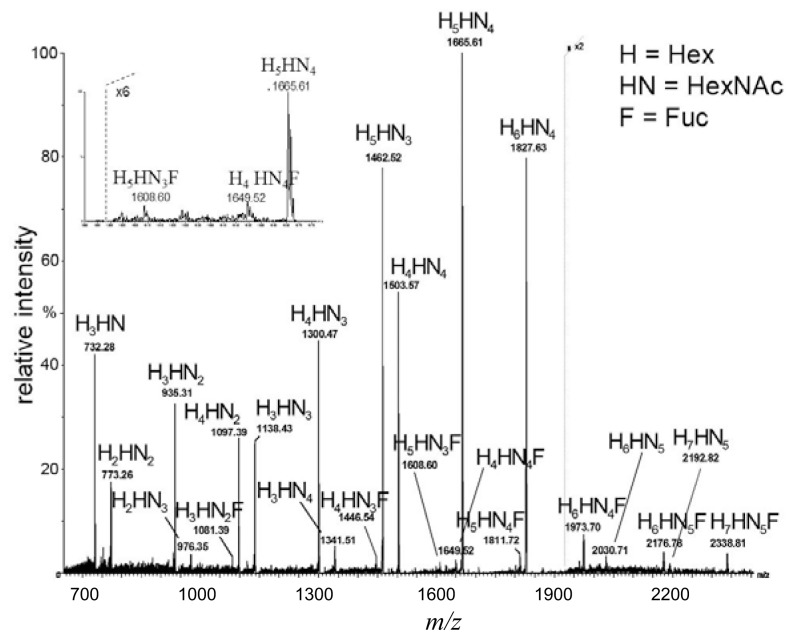
The (+) matrix-assisted laser desorption/ionization (MALDI) quadrupole-time-of-flight (Q-TOF) spectrum of the sample containing human milk oligosaccharide alditols. The *m*/*z* values represent [M + Na]^+^ ions.

The MALDI TOF map of the sample containing human milk oligosaccharides is shown in Figure 1. Twenty two different ions were detected that could be assigned according to monosaccharide compositions (in terms of the building blocks, Hex (H, hexose), HexNAc (HN, *N*-acetylhexosamine) and dHex (F, deoxyhexose)). The oligosaccharides ranged from tetra- to dodeca-saccharides, including eight fucosylated structures. The MALDI spectrum was acquired from the sample without further purification procedures as an initial oligosaccharide mapping experiment. The most intense ions represent octa-, nona- and deca-saccharides. The undeca- and dodeca-saccharide-related ions were at a lower intensity. Tetra- to hepta-saccharide series were detected, as well, with similar relative abundances.

### 2.2. De Novo MALDI Q-TOF CID Data Analysis Using Diagnostic Ions

**Figure 2.**
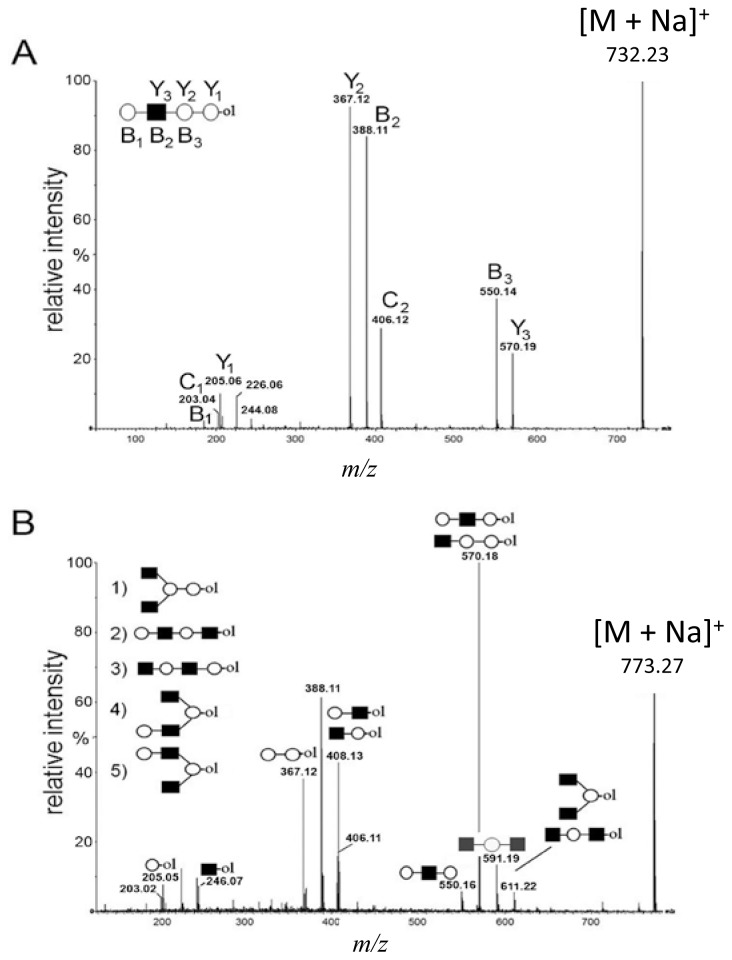
The (+) MALDI Q-TOF collision-induced dissociation (CID) spectra of two low mass oligosaccharide alditol ions from the mixture (Figure 1). (**A**) Tetrasaccharide alditol lacto-*N*-tetraose (732.23); and (**B**) isobaric mixture of tetrasaccharide alditols (773.27) of the sum composition Hex_2_HexNAc_2_.

The fragmentation spectra (MS/MS) were assigned using the theoretical cleavage ions according to Domon and Costello [23]. In Figure 2A, the CID spectrum of the basic tetrasaccharide precursor ion at *m*/*z* = 732 is shown. Due to the reduced reducing end, it was possible to unambiguously assign Y_1–3_ ions for the full sequence information, along with B_1–3_ ions (*m*/*z* = 185, 388 and 550), in agreement with the known biosynthetic core structure of human milk oligosaccharides (HMO) belonging to LNT and LNnT tetrasaccharides. No intra-ring fragmentation was observed under these conditions.

In Figure 2B, the CID spectrum of parent ion at *m*/*z* = 773 assigned to H_2_HN_2_ is shown. The cleavage ions at *m*/*z* = 205, 246, 367, 408, 550, 570, 591 and 611 are postulated to be the partial structures shown. From the ion at *m*/*z* = 367, Structure 1 could be postulated. For the ion at *m*/*z* = 408, two tetrasaccharide structures (2 and 3) are possible, according to HMO biosynthetic rules. Structure 2 corresponds to the ion at *m*/*z* = 246. Structure 3 was not confirmed, due to the ambiguity of fragment ions at *m*/*z* = 205 and 570, which could also be generated from Structure 1. It is important to note that both Structure 2 and 3 could have been built upon enzymatic or chemical hydrolysis of HMO. Furthermore, both Structures 4 and 5 could give rise to fragment ions at *m*/*z* = 408 and 611.

In Figure 2, MS analysis of oligosaccharides when isomeric mixtures are present is presented. For manual *de novo* tandem MS data analysis, the pragmatic first step is the assignment of diagnostic ions (Table 1). Accordingly, the fragment ion at *m*/*z* = 367 as a diagnostic one is relevant for Structure 1, indicating the presence of the lactose core on the reducing end. The fragment ion at *m*/*z* = 246 is diagnostic for the presence of HexNAc on the reducing end, typical for the truncated structure. The diagnostic ion at *m*/*z* = 408 in combination with other diagnostic ions, such as at *m*/*z* = 205 (indicating the presence of a hexose on the reducing end) and at *m*/*z* = 246 (indicating the presence of an *N*-acetylhexosamine on the reducing end), represents the “conditional” diagnostic ion, because additional information was necessary to fully assign its structure. In the case of isomeric mixtures, this information can be probed with MS^3^ experiments on appropriate instrument types for full structural elucidation. A list of diagnostic and conditional diagnostic ions used in this study is shown in Table 1. Ions at *m*/*z* = 246, 367 and 794 in Table 1 are diagnostic ones. Additionally, two examples of non-diagnostic ions are given, illustrating that no significant information can be deduced from them, alone or in combination with other fragment ions.

Efficient fragmentation has been obtained by MALDI Q-TOF CID (Micromass, Manchester, UK) from major parent ions at *m*/*z* = 732, 773, 935, 1097, 1138, 1300, 1462, 1503, 1665 and 1827. The parent ion at *m*/*z* = 1341 fragmented poorly. The parent ion at *m*/*z* = 1503, was further tested along low-intensity parent ions at *m*/*z* = 1608, 1973 and 2338 on an AB SCIEX QSTAR^®^ Pulsar i instrument (AB SCIEX, Toronto, ON, Canada).

**Figure 3.**
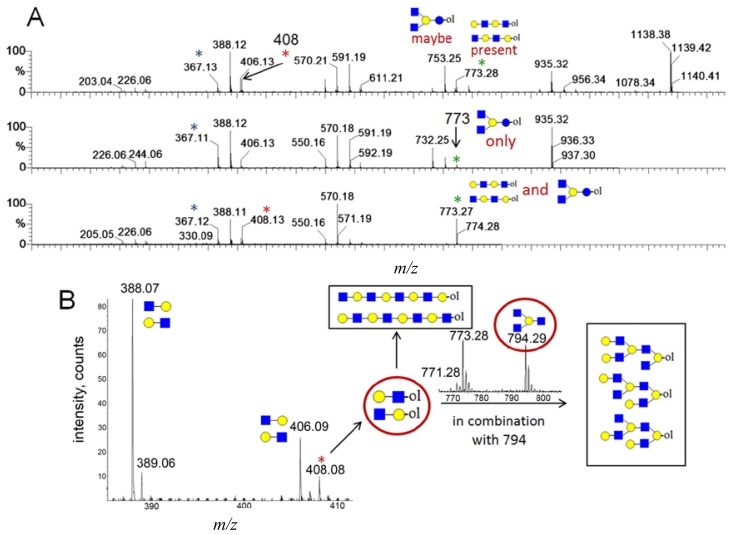
The assignment of CID MS data using diagnostic (367 and 794) and conditional diagnostic (408 and 773) fragment ions. (**A**) Ions at *m*/*z* = 773, 935 and 1138; and (**B**) zoom-in of the mass range of 385–410 and 770–800 Da. Possible structures are all in accord with the fragmentation pattern. 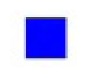 denotes *N*-acetylglucosamine, 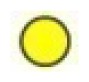 galactose and 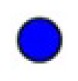 glucose. 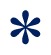, 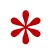 and 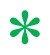 denote ions relevant to the explanation in the text.

In Figure 3A, the fragmentation spectra of parent ions at *m*/*z* = 773, 935 and 1138 are depicted. A structural assignment has been carried out using the diagnostic ion at *m*/*z =* 367 (blue asterisk) and conditional diagnostic ions at *m*/*z* = 408 (red asterisk) and 773 (green asterisk) (see Table 2). For the parent ion at *m*/*z* = 935, the assignment to two possible isomeric pentasaccharide structures is ambiguous. Based on the diagnostic ion at *m*/*z* = 367 and the conditional diagnostic ion at *m*/*z* = 773 (which in the absence of the conditional diagnostic ion at *m*/*z* = 408 and the presence of the diagnostic ion at *m*/*z* = 367 indicates the presence of a branching point at Hex_2_ (see Table 2)), the presence of a single tetrasaccharide pattern (structure type) could be postulated. However, the location of the final galactose residue cannot be deduced from our data. Furthermore, the presence of a linear pentasaccharide structure cannot be proven in the presence of branched structure(s). Finally, in the case of the CID of parent ion at *m*/*z* = 1138, we detected all three ion types at *m*/*z* = 367, 408 and 773. According to the diagnostic ion at *m*/*z* = 794, a branching point is at Hex_4_. A branching point at Hex_2_ (supported by the presence of the conditional diagnostic ion at *m*/*z* = 773) could not be deduced due to the presence of another conditional diagnostic ion at *m*/*z* = 408.

In Figure 3B, the inserts of the MALDI Q-TOF CID spectrum of the parent ion at *m*/*z* = 1503 are shown, obtained by using the “enhance all” function on the AB SCIEX QSTAR^®^ Pulsar i (see the Experimental Section). In this experiment, the conditional diagnostic ion at *m*/*z* = 408 was detected (left insert) and allowed one to deduce the presence of two further oligosaccharide structures in the *m*/*z* = 1503 parent ion mixture. Furthermore, in combination with the diagnostic ion at *m*/*z* = 794, the existence of three additional truncated isomeric structures could be proposed (if we restrict our analysis to the reported HMO structures, up to lacto-*N*-decaose), but even more isomeric structures are possible, when Type I and Type II branches are considered [19]. Therefore, at least five novel structure types could be added to the list of possible isomeric octasaccharide structures present in the parent ion at *m*/*z* = 1503, most of them as truncated isomers.

### 2.3. Analysis of Branched Higher Molecular Weight Oligosaccharides

**Figure 4.**
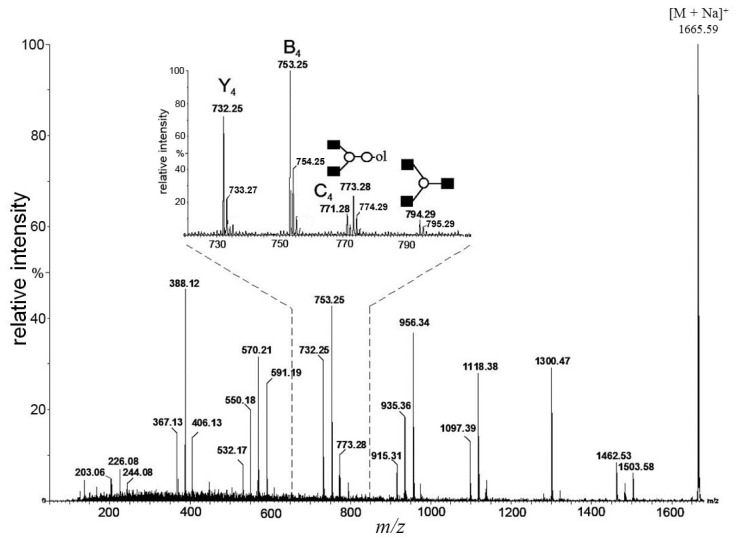
Fragmentation spectrum of the parent ion at *m*/*z* = 1665.59. The analysis of fragment ions revealed the presence of isobaric nonasaccharide alditols.

In Figures 2 and 3, we showed how the existence of truncated structures can exponentially increase the number of theoretically possible isomers present in the isobaric parent ion mixture. In Figure 4, the MALDI Q-TOF CID spectrum of the parent ion at *m*/*z* = 1665 and the conditional diagnostic ion at *m*/*z* = 408 was not present, but the positive structural evidence was obtained by two important fragment ions, the conditional diagnostic ion at *m*/*z* = 773 and the diagnostic ion at *m*/*z* = 794. The diagnostic ion at *m*/*z* = 367 together with the conditional diagnostic ion at *m*/*z* = 773 provides evidence of branched nonasaccharide structures with a branching point at Hex_2_. The diagnostic ion at *m*/*z* = 794 indicates the presence of branched nonasaccharide structures with a branching point at Hex_4_. By applying biosynthetic rules and restricting our analysis to the reported HMO structures (up to lacto-*N*-decaose), only three out of 13 theoretical branched structure types remain possible.

### 2.4. MALDI Q-TOF CID of Low-Intensity and High Molecular Weight Parent Ions

**Figure 5.**
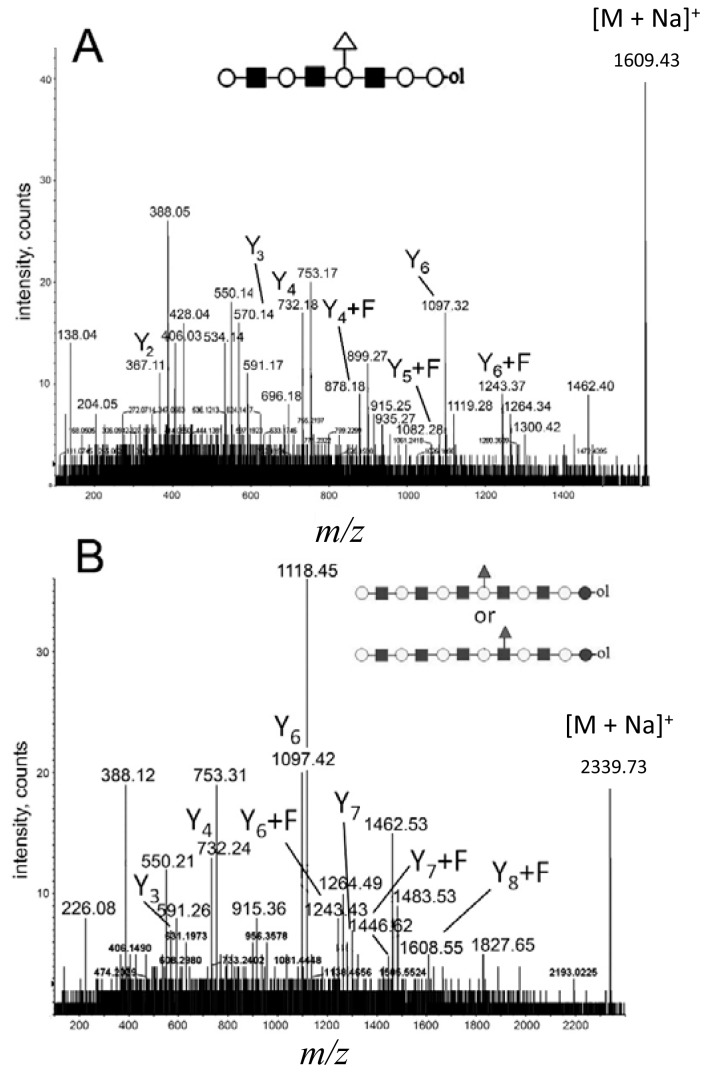
The potentials and limits of fragmentation data using low abundant ions at high *m*/*z* values. (**A**) Parent ion at *m*/*z* = 1608.43; and (**B**) parent ion at *m*/*z* = 2338.73. The spectra were acquired on the AB SCIEX QSTAR^®^ Pulsar i (see the Experimental Section).

The ability to sequence high molecular weight and low-intensity precursor ions with MALDI Q-TOF CID is illustrated in Figure 5, where the CID spectra of parent ions at *m*/*z* = 1608 (Figure 5A) and 2338 (Figure 5B) are shown. The precursor ion at *m*/*z* = 1608 is of low abundance, and the precursor ion at *m*/*z* = 2338 is the highest *m*/*z* ion in the primary mixture (Figure 1); both are fucosylated. Both low abundant precursor ions delivered on the AB SCIEX QSTAR^®^ Pulsar i, with the “enhance all” function enabled, good evidence and sufficient sequence information to deduce linear octasaccharide and dodecasaccharide HMO chains. Furthermore, a significant amount of fucosylated product ions were detected. We were able to clearly assign the fucosylation site to Hex_4_ in the case of fucosylated octasaccharide (Figure 5A). In the case of fucosylated dodecasaccharide (Figure 5B), fragment ions corresponding to either Y_5_ or Y_5_ + F ions were not detectable, while the Y_6–8_ + F ion series was visible. Accordingly, the position of the fucose residue in the dodecasaccharide HMO structure was not assigned, indicating the limits of sensitivity at sequencing low intensity, high molecular weight oligosaccharides.

### 2.5. Analysis of HMO Truncated Structures

**Figure 6.**
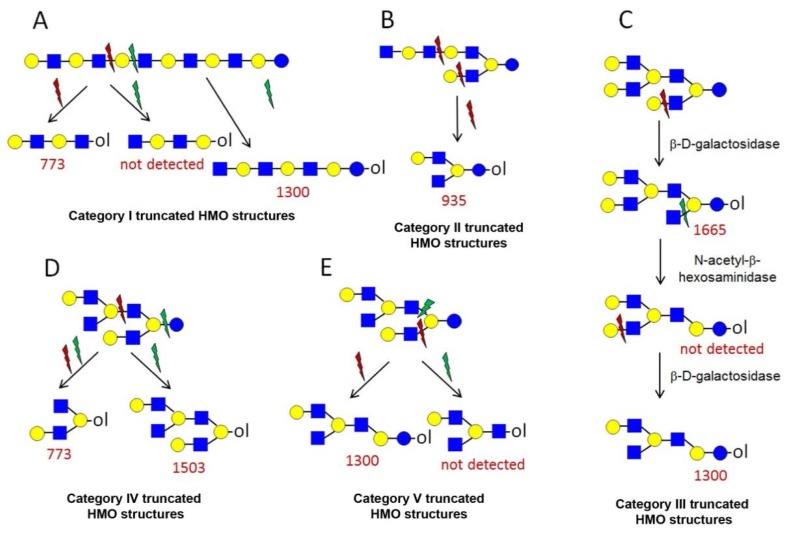
The formation of truncated oligosaccharide structures in the human milk oligosaccharide (HMO) fraction. 
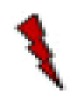
 and 
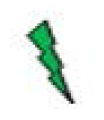
 depict various possible hydrolysis events, each of which, alone (**A**–**D** right, **E**) or combined (**D** left), yield different oligosaccharide structure types shown in the figure. 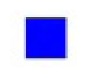 denotes *N*-acetylglucosamine, 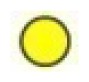 galactose and 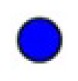 glucose.

A number of oligosaccharides have been identified as truncated structures by their MS/MS patterns. In Figure 6, the possible mechanism of the generation of such structures is presented. The proposed reaction schemes in five practical categories (I–V) for linear and branched species is illustrated, following single or multiple truncation, based on fragmentation data and the biosynthetic considerations of the elongation of the lactose core by *N*-acetyllactosamine units in a linear or branched fashion. Most evident are oligosaccharide structures with an odd monosaccharide composition, which could possibly arise also by the exoglycosidase cleavages [6].

Category I (Figure 6A) is the simplest type of hydrolysis. The resulting oligosaccharides would give rise to a fragment ion at *m*/*z* = 408. Furthermore, these hydrolytic products could all give rise to odd-numbered linear oligosaccharide structures. Similar to Category I, the same hydrolytic events could occur on branched oligosaccharide structures, giving rise to odd-numbered Category II branched oligosaccharide structures (Figure 6B). Category III represents possible exoglycosidase events, described previously [6]. The sequential trimming of monosaccharide units on the nonreducing termini can yield structures, such as H_5_HN_4_ (*m*/*z* = 1665), which is the most abundant component in our sample (see Figure 1). In the case of the nonasaccharide, the trimming of another terminal galactose residue seems favourable, leading to respective H_4_HN_4_ structures, while the trimming of a *N*-acetylglucosamine (GlcNAc) residue seems unfavourable or leads to a quickly degradable product. Category IV hydrolysis depicts the type of structures arising from hydrolytic events on the nonreducing end of glucose. All parent ion mixtures that gave rise to the fragment ion at *m*/*z* = 611 could theoretically contain these type of structures (parent ions at *m*/*z* = 773, 1138 and 1503), although they seem to be present in very low amounts. Category V hydrolysis shows the remaining two theoretical hydrolytic fragments. We detected some of these types of fragments unambiguously in parent ions at *m*/*z* = 1138 and 1300, albeit they were present only near the detection limit in the latter.

### 2.6. Fragmentation of the Ion at m/z = 1341

The assignment of the parent ion at *m*/*z* = 1341.51 is based on the fragment ions at *m*/*z* = 794 and 773 (spectrum not shown). This type of branching pattern has been described before, but is still considered novel [2,24], perhaps due to its incomplete characterization and rare observance in the published literature. One novel and five additional possible HMO structures were postulated recently to be based on this structure [2]. This structure type has been indeed resolved previously by FAB (fast atom bombardment) MS and NMR spectroscopy on an example of lacto-*N*-decaose in 1988 [20], as reviewed recently [25]. Having proven the existence of this branching pattern in our sample and HMO mixtures, in general, it is indicated as a possible isomer in the isobaric parent ion mixtures containing the diagnostic fragment ion at *m*/*z* = 794. It would be interesting to prove the existence of these isomers in the high molecular weight HMO fractions and to determine the relative abundance of this branching pattern in HMO in general. One line of study in this direction has been carried out by Amano *et al*. [19], in which the decaose structure has been best described so far in terms of its fucosylation patterns.

### 2.7. General Conclusions about Identified HMO Structures

After having analysed all the CID data, it became possible to summarize the data (Table 2), as well as to categorize the postulated structures, depending on the monosaccharide composition of oligosaccharides (Table 3). Accordingly, all HMO with the general formula H*_n_*_+2_HN*_n_* were identified as linear. The odd-numbered HMO with the general formula H*_n_*_+1_HN*_n_* were all truncated on the nonreducing termini only. The HMO with the general formula H*_n_*HN*_n_* were most diverse. Unlike in the first two groups, this group always contained the fragment ion at *m*/*z* = 408, indicative of truncation on the reducing termini. They also had multiple truncations on the non-reducing termini. Surprisingly, they are not the most abundant structures in the spectrum and are only about half as abundant as the previous two groups. Finally, the HMO of the general formula H*_n_*_−1_HN*_n_* were of very low abundance. They also yielded the *m*/*z* = 408 fragment ion, although it could not be detected for H_3_HN_4_, most likely due to the low intensity of fragment ions obtained in the CID spectrum. Monofucosylated parent ions corresponding to all these groups were detected, except for the H*_n_*_−1_HN*_n_* group. Further and more detailed studies will be carried out to study these phenomena. In particular, quantitative analysis should provide important insight about the abundance of individual HMO isomers.

## 3. Experimental Section

A pool of human milk was obtained from Donor 0, a Le^a^ non-secretor. The high molecular weight chromatographic fraction was obtained by gel permeation Biogel P-4 chromatography and submitted to mild hydrolysis to remove fucose and sialic acid, as described in Bruntz *et al*. [20]. The oligosaccharide fraction was reduced with NaBH_4_ and analysed using MALDI MS in the positive ion mode.

2,5-Dihydroxybenzoic acid (DHB) (Sigma, Steinheim, Germany) was used as a MALDI matrix. For MALDI TOF analysis, γ = 20 mg/mL, dissolved in H_2_O/ACN (acetonitrile) 1:1 (*v*/*v*), was used. For MALDI Q-TOF analysis, γ = 80 mg/mL in H_2_O/ACN 70%:30% (*v*/*v*) was used. The oligosaccharide sample was dissolved in ddH_2_O, and 1 μL of the sample was mixed with 1 μL of matrix solution on the MALDI target and allowed to dry. MALDI TOF MS experiment was performed on a TofSpec 2E instrument (Micromass, Manchester, UK), equipped with a LeCroy (Teledyne Lecroy Inc., New York, NY, USA) digitizer LSA1000 with a 2 GHz acquisition rate. The majority of the MALDI Q-TOF CID experiments were conducted on a prototype Micromass instrument. Additional MALDI Q-TOF CID experiments were performed on a commercial instrument, AB SCIEX QSTAR^®^ Pulsar i.

In general, there are differences in experimental parameters between these three instruments, as axial (MALDI TOF) and orthogonal (MALDI Q-TOF) instruments have different modes of operation. For MALDI TOF MS, the minimum laser fluence that allowed stable signal collection was used, in order to increase the spectral resolution, to minimize possible detector saturation and to minimize in-source and post-source decay (ISD and PSD). For the prototype MALDI Q-TOF instrument from Micromass, as well as the commercial AB SCIEX QSTAR^®^ Pulsar i, no sample-to-sample signal optimization was required, as the instrument default method was robust enough. The practical difference in the mode of operation between MALDI TOF and MALDI Q-TOF instruments is the high laser fluence requirement for MALDI Q-TOF instruments. This is technically necessary because orthogonal instruments are less efficient in the ion transport than axial instruments, and therefore, more ions need to be produced within the oMALDI (orthogonal MALDI) source. Two practical consequences of higher laser fluence used in our hands were: (i) the ability to use higher γ(DHB) as a matrix solution on MALDI Q-TOF instruments; and (ii) the use of DHB as a matrix was required, as other matrices used in our laboratory did not give any signal on a MALDI Q-TOF instrument. Despite these technical and experimental differences, the MS1 spectra on all three instruments were very similar. However, in our hands, the MALDI TOF oligosaccharide map of the analysed chromatographic fraction acquired on the Micromass TofSpec 2E was of slightly higher quality in terms of signal intensity and is, thus, presented here.

MS/MS spectra were obtained by collisionally-induced dissociation (CID) using Ar as a collision gas on MALDI Q-TOF instruments under conditions like low-energy CID. The collision energy that produced the optimal fragmentation pattern across the whole *m*/*z* range for a given parent ion was adjusted to be proportional to the parent ion *m*/*z* value. The AB SCIEX QSTAR^®^ Pulsar i instrument has a unique additional mode of operation in MS/MS mode, in which it can amplify the intensity of the detected fragment ions by using a “pulse” function option, which allows the user to select which *m*/*z* range in the CID spectrum needs to be “enhanced” (in terms of fragment ion intensity). When this option is chosen, the built-in control software automatically recalculates “pulsing” windows, which determine which fragment ion packets are sent to the TOF analyser more often, thus increasing their relative intensities in the final CID spectrum. The “enhance all” option in the control software allows enhanced product ion detection over the whole *m*/*z* range of the CID spectrum. This option in the recording of the CID spectra of low-intensity parent ions was functional (Figure 5), as well as in the recording of diagnostic low-intensity fragment ions (Figure 3B).

## 4. Conclusions

*De novo* analysis of tandem MS data is described as an option for an in-depth view on already known or new structures. Since many molecular ions represented isomeric mixtures of oligosaccharides, a rationale for the assignment based on the biosynthetic pathway was applied. Oligosaccharide structures not found in databases were proposed for assignment according to specific diagnostic ions detected in fragmentation spectra. In the mixture of linear and branched HMO structures within a single precursor ion *m*/*z* value, the isomeric components were assigned using a diagnostic ion analysis technique. A minor amount of fucosylated oligosaccharides was found, where fucosylation occurred on a single site. In summary, four non-fucosylated linear HMO and three monofucosylated linear HMO were characterized. About twenty branched structure types were proposed, as in agreement with MS/MS data, known HMO structures and hydrolytic patterns. These structures are furthermore isomeric with many possible biosynthetic HMOs and, when taking into account the 1–3 and 1–4 galactose linkage isomers, over a hundred HMO structures can be assumed to be present in the analysed chromatographic fraction. The summarized results are depicted in Tables 2 and 3. Truncated structures without glucose are omitted for clarity.

Although we were able to determine the presence of isomeric structures in our sample, our data also illustrate the limitations of MALDI Q-TOF CID technique in their full characterization. It is important to note that mass spectrometry in general can overcome these limitations in several ways: (i) by using an additional liquid chromatography (LC) or capillary electrophoresis (CE) separation step prior to MS analysis; (ii) by using high-energy CID to generate cross-ring cleavages; and (iii) by using MS^n^ fragmentation techniques. Additional LC separation step prior to MALDI Q-TOF MS/MS analysis can be done in off-line mode [26]. In on-line mode, the applicability of LC-ESI-MS/MS [2,3,17] and CE-LIF (laser induced fluorescence)-MS^n^ [16] in the analysis of HMO has been well documented. Unlike low-energy CID, used in MALDI Q-TOF instruments, high-energy CID used in MALDI TOF-TOF instruments typically yields cross-ring cleavages, which can carry information about the branching pattern of the parent ion [14,15]. Finally, the MS^n^ technique has been successfully applied to the analysis of HMO [19]. In general, different MS instrumentation setups are required for obtaining the maximum amount of structural information about the analysed sample and these often require either more time for analysis (LC-MS, CE-MS, MS^n^), a derivatization step prior to MS analysis (CE-MS, MS^n^) [16,19,27] and more time or additional software tools to analyse the large amount of data these techniques yield [2,18]. Traditionally, MALDI MS has been advantageous in the high-throughput MS1 screening of biological samples, and with modern instrumentation allowing the acquisition of high-quality MALDI MS/MS data, this property of MALDI MS can be extended to the high-throughput screening of tandem MS data, as well.

The advantages of MALDI Q-TOF CID for structural elucidation of complex oligosaccharides are the precursor ion selection properties, the ability to sequence very low abundant parent ions and the speed of the MS/MS data acquisition. The majority of MS1 signals could be sequenced with the prototype Micromass MALDI Q-TOF instrument, and all detectable signals could be sequenced with the commercial AB SCIEX MALDI Q-TOF instrument. This technical advancement alone allows for a huge jump in the data throughput of a glycomic research laboratory, where MALDI MS is the method of choice for all preliminary experiments related to the structural characterization of complex oligosaccharide mixtures. Finally, the advantages of the MALDI Q-TOF MS/MS indicate its applicability to high-throughput sample analysis, requiring new software tools for faster data analysis [18,28].

Future studies on high molecular weight HMO should provide better insight on biologically interesting data, like the quantitation of isomers and the reexamination of sequences, which indicate the biosynthetic pathways involved. Quantitative studies are necessary in order to determine the relative abundance of particular isomers, *i.e.*, are they all present in equal, random or more selective amounts, where the presence of certain isomers can be neglected.

## Figures and Tables

**Table 1.** Diagnostic and conditional diagnostic ions found and monosaccharide symbols used in this study.

Type of ions	*m*/*z*	Type	Structure	
non-diagnostic ions	753	B_4_ or internal B/Y fragment ion	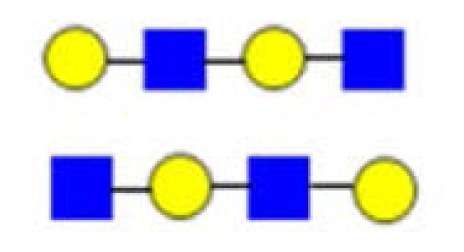	
	771	C_4_ or internal C/Z fragment ion	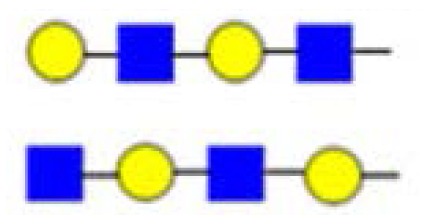	

diagnostic ions	205	hexose at reducing end	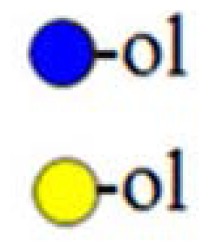	
	246	HexNAc at reducing end (non-biosynthetic)	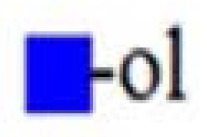	
	367	contains biosynthetic core lactose unit	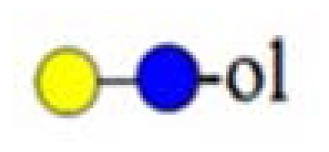	
	794	branching at Hex_n≥4_	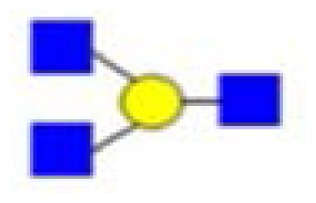	

conditional diagnostic ions	408	non-biosynthetic reducing end structure	no 246	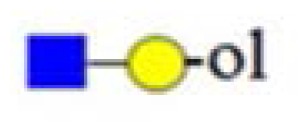
			no 205	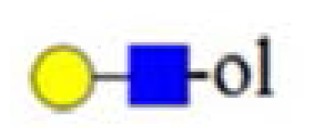
	611	non-biosynthetic reducing end structure	no 246	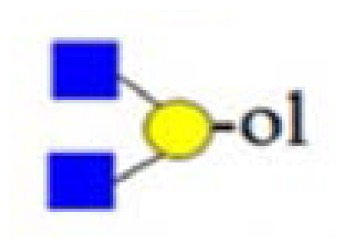
			no 205	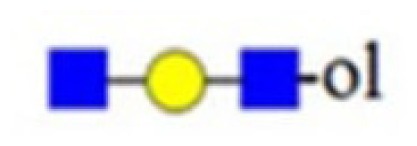
	732	Y4 with core lactose—linear oligosaccharide	no 773 and no 794	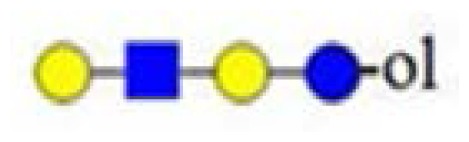
	773	branching at Hex_2_ (biosynthetic structure)	yes 367 and no 408	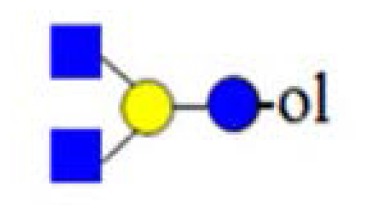

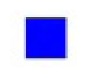
 denotes *N*-acetylglucosamine, 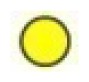 galactose and 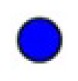 glucose.

**Table 2.** Confirmed and proposed oligosaccharide alditol structures along with respective diagnostic ions. Additional branched isomers are possible, due to galactose 1–3 and 1–4 linkages, and most of them are further fucosylated *in vivo* with fucose 1–2, 1–3 or 1–4.

Monosaccharide composition	Biosynthetic	Truncated with lactose core	Truncated without lactose core	Diagnostic ions
H_3_HN (732)	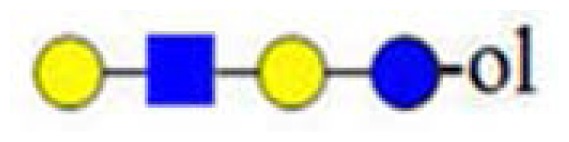	-	-	367
H_2_HN_2_ (773)	-	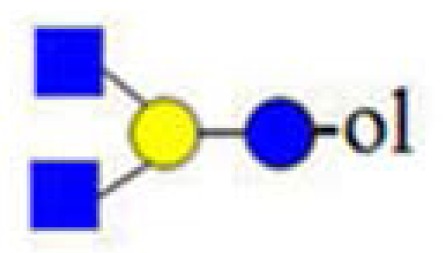	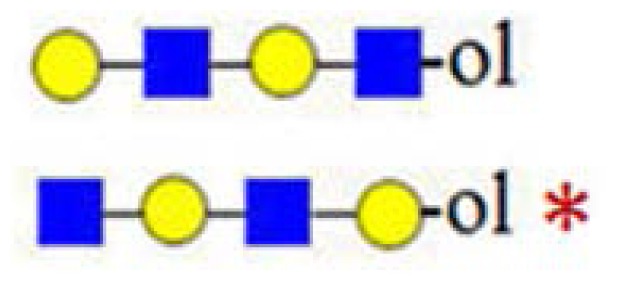	246, 367, 408
H_3_HN_2_ (935)	-	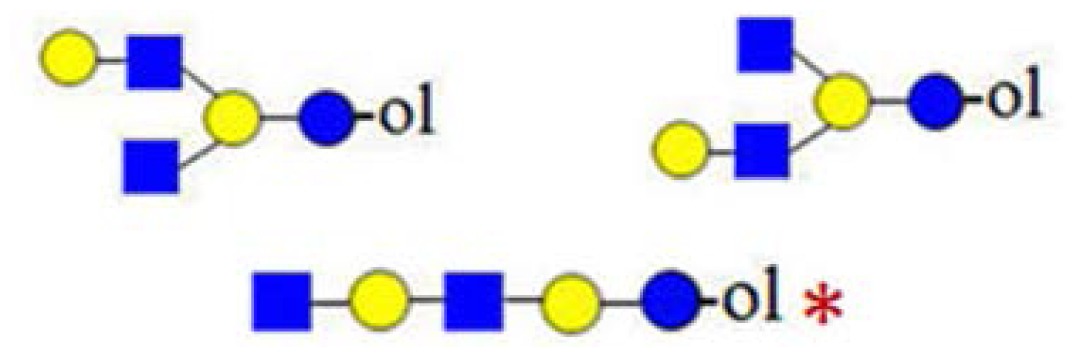	-	367, 773
H_2_HN_3_ (976)	-	-	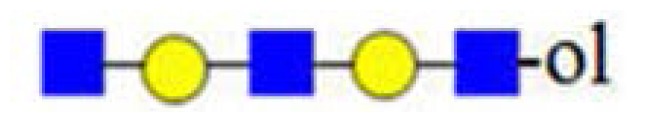	-
H_4_HN_2_ (1097)	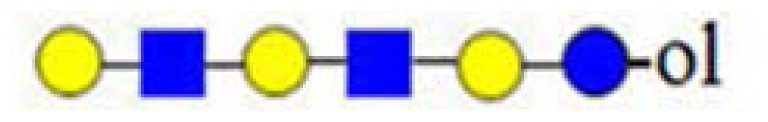	-	-	367
H_3_HN_3_ (1138)	-	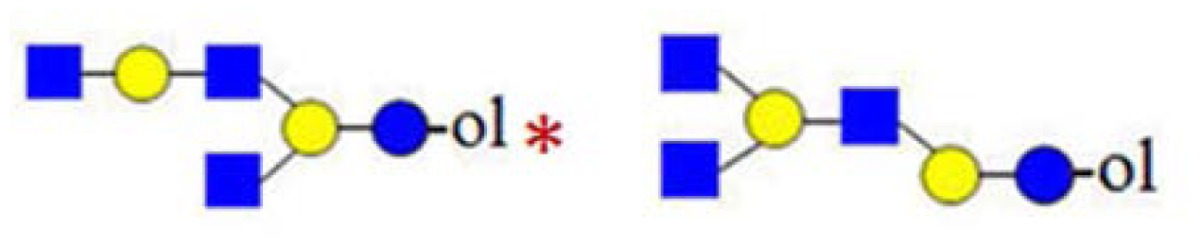	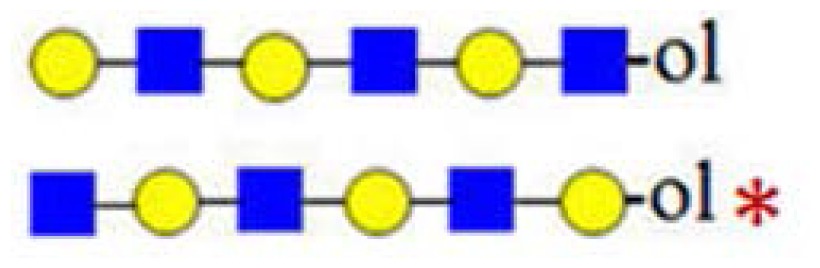	246, 367, 408, 773, 794
H_4_HN_3_ (1300)	-	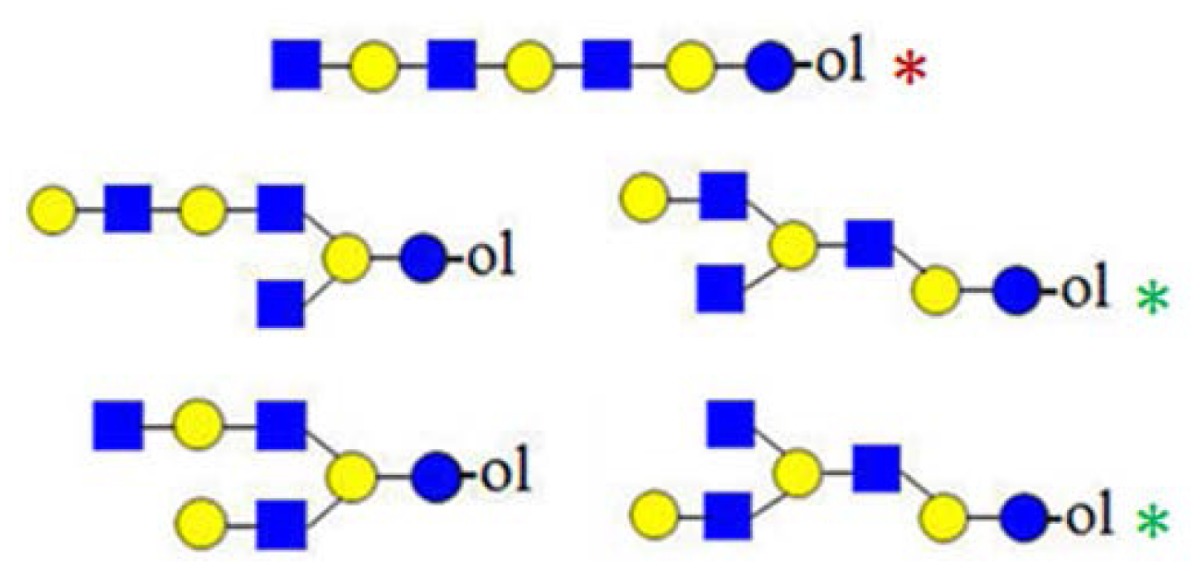	-	367, 773, 794
H_3_HN_4_ (1341)	-	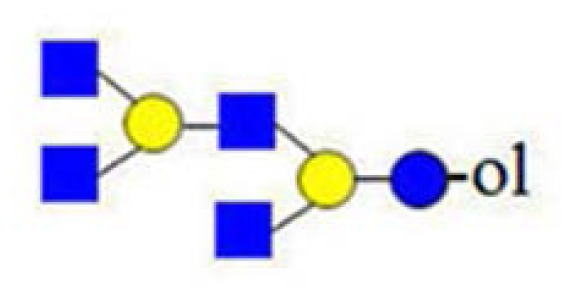	-	773, 794
H_5_HN_3_ (1462)	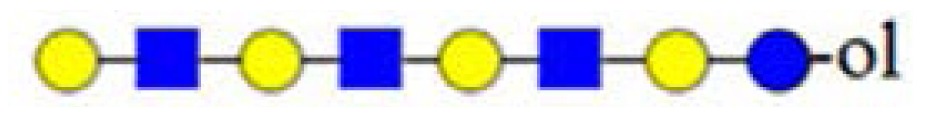	-	-	367
H_4_HN_4_ (1503)	-	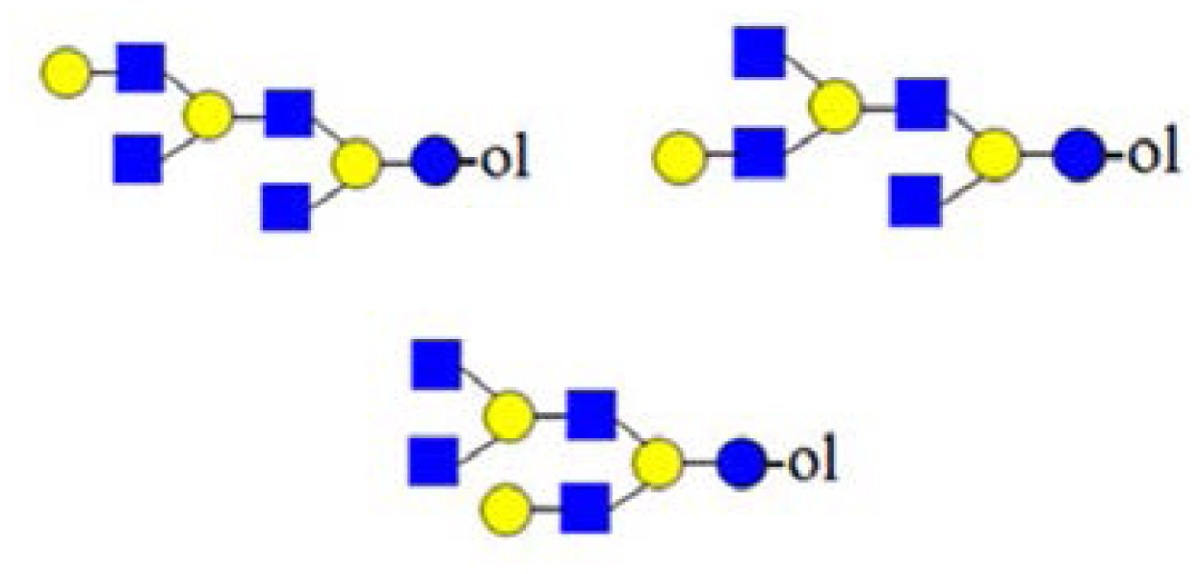	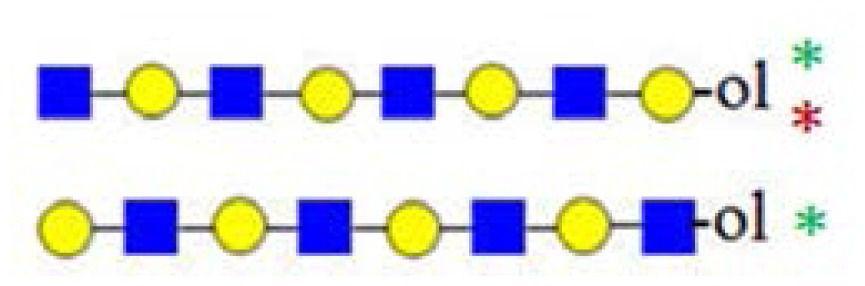	367, 408, 773, 794
H_5_HN_4_ (1665)	-	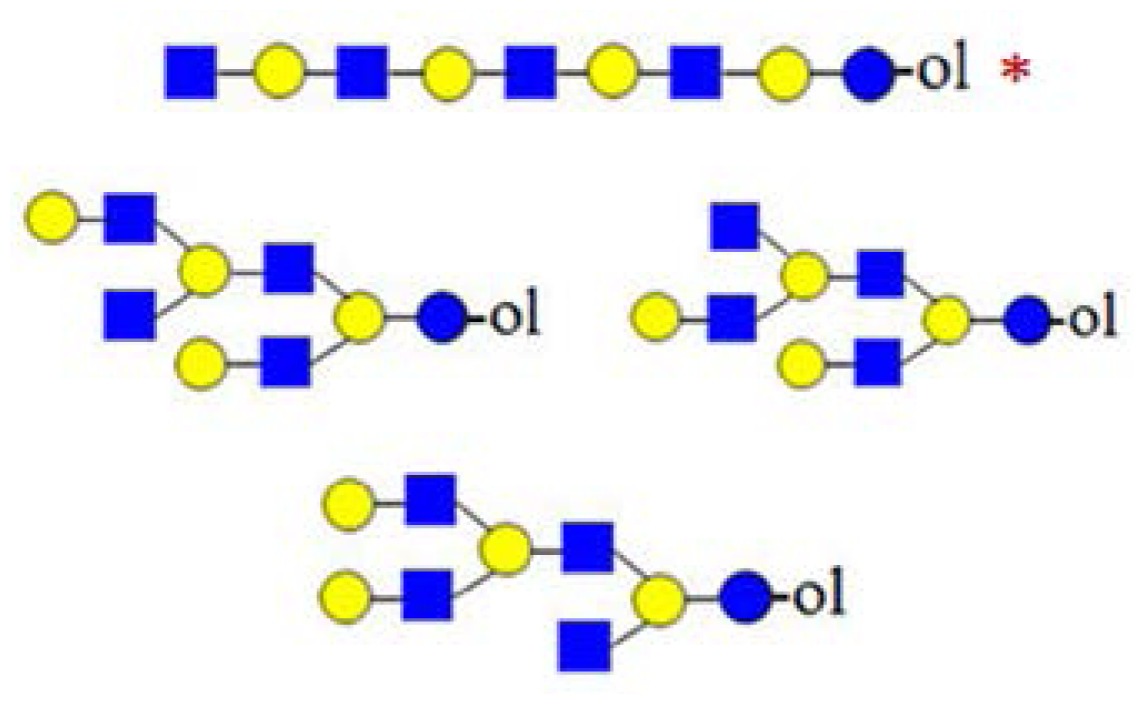	-	367, 773, 794
H_6_HN_4_ (1827)	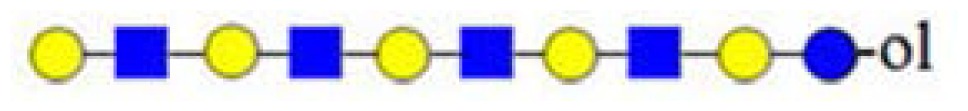	-	-	367
H_5_HN_3_F (1609)	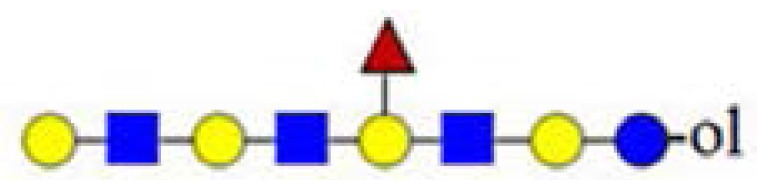	-	-	878
H_6_HN_4_F (1973)	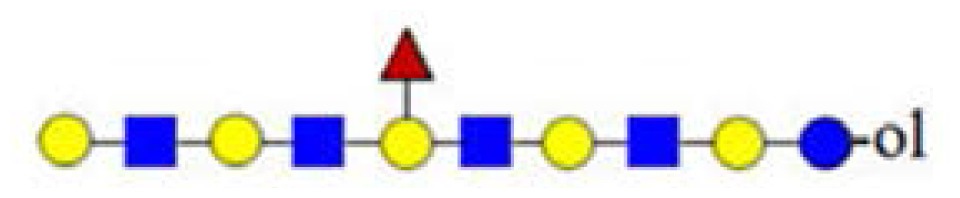	-	-	1243
H_7_HN_5_F (2338)	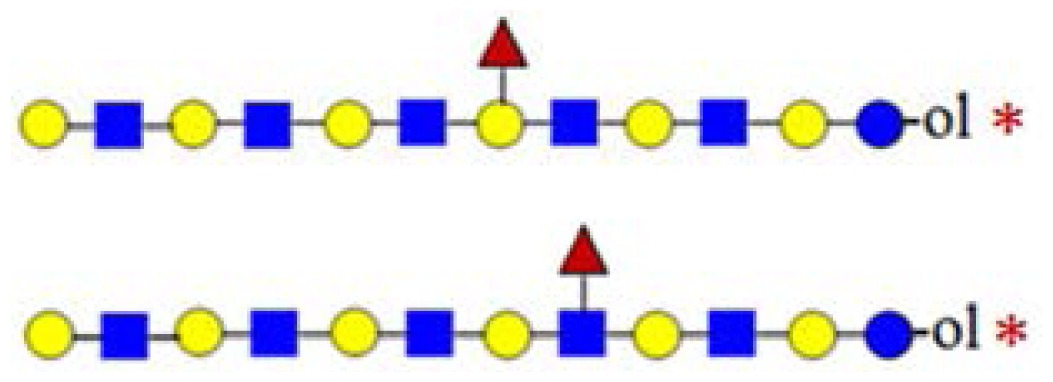	-	-	1243 ?

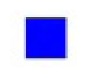
 denotes *N*-acetylglucosamine, 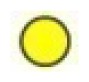 galactose, 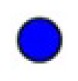 glucose and 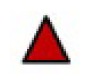 fucose.

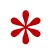
 structures that could be neither confirmed nor excluded.

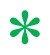
 present in very small amounts.

**Table 3.** Categorization of HMO structures identified in this study based on their monosaccharide composition.

Monosaccharide composition	HMO structure types identified
H_n+2_HN_n_	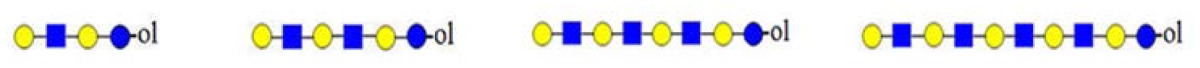
H_n+1_HN_n_	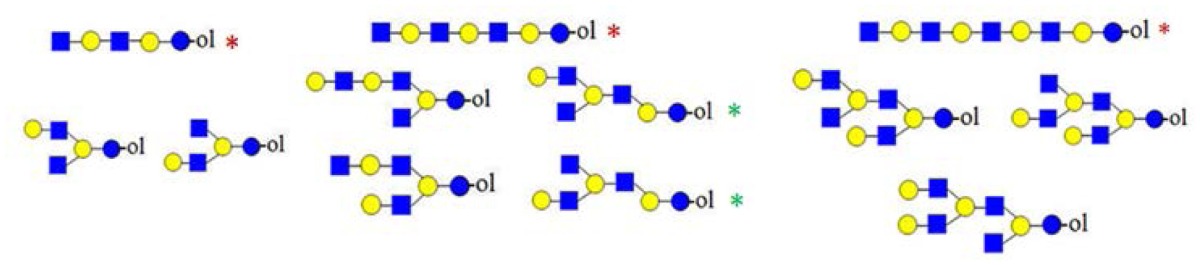
H_n_HN_n_	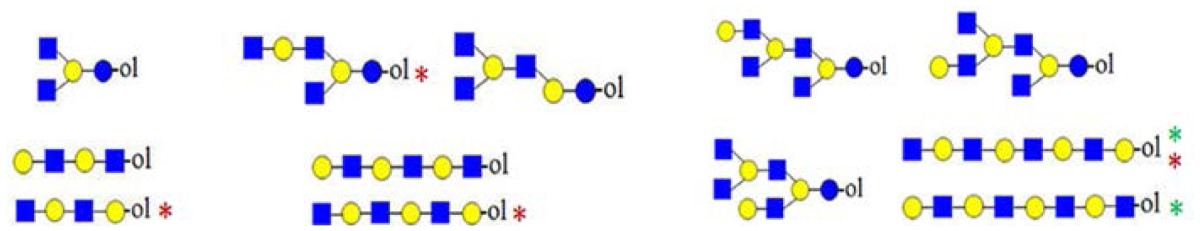
H_n−1_HN_n_	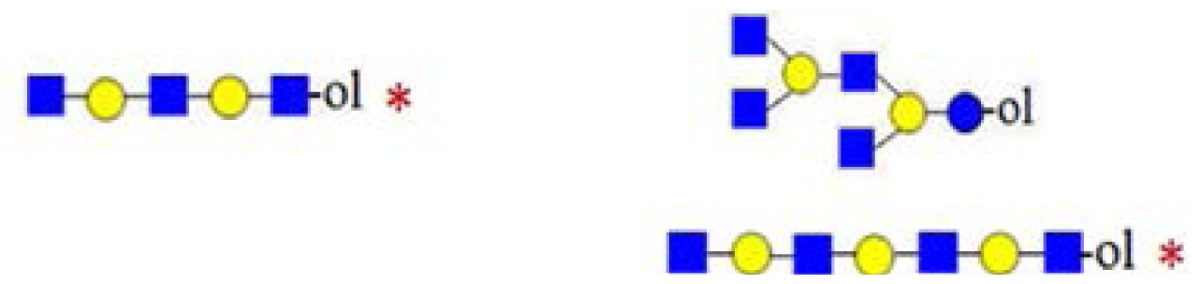

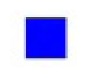
 denotes *N*-acetylglucosamine, 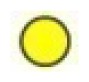 galactose and 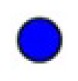 glucose.

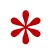
 structures that could be neither confirmed nor excluded.

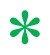
 present in very small amounts.
